# Empathy strengthens the effect of emotion on beauty

**DOI:** 10.21203/rs.3.rs-4920639/v1

**Published:** 2024-09-11

**Authors:** Anna Bruns, Denis G. Pelli

**Affiliations:** New York University; New York University

## Abstract

Past research shows that emotion affects beauty judgments of images and music. Because it is widely supposed that our faculty of empathy facilitates aesthetic experience, we wondered whether individual levels of empathy modulate the effect of emotion on beauty. 164 participants rated the perceived beauty, happiness, and sadness of 12 art images, 12 nature photographs, and 24 songs. The stimuli were presented in two blocks, and participants took the PANAS mood questionnaire before and after each block. Between blocks, they viewed one of three mood induction videos, intended to increase their happiness, increase their sadness, or leave their mood unchanged. We also measured (trait) empathy with the Questionnaire for Cognitive and Affective Empathy. We used structural equation modeling to analyze the effect of empathy on emotion, beauty, and the relationship between them. We assessed four emotion variables: participants’ felt happiness and sadness (mood questionnaire ratings) and perceived happiness and sadness (stimulus ratings). We find that higher empathy is associated with stronger positive relationships between beauty and both felt and perceived emotions, for both images and music (*β* ~ 0.06 per empathy point on a 10-pt. scale, *p* < 0.001). We also find that perceived happiness and sadness boost beauty directly for both images and music. However, sadness affects music more than images (*β* = 0.51 vs. 0.12, all *p* < 0.001), and empathy amplifies this relationship for music but not images. Thus, felt and perceived emotions produce more beauty, more so in more empathic people, and more so with music than images.

## Introduction

When you listen to a song, read a poem, or look at a face, what determines whether or not you will find it beautiful? Empirical aesthetics researchers generally approach this question in one of two ways: by studying properties of the object – the piece of music, poem, the face – or qualities of the observer – their cultural context, their art expertise, their personality (Brielmann & Pelli, 2018). In the present study we attend to both object and observer qualities, and the quality of interest is emotion: emotion perceived in the object (“perceived emotion”) and emotion felt by the observer (“felt emotion”). Specifically, we ask: how do perceived and felt emotions affect beauty judgment? Do we find objects that express certain emotions especially beautiful? Do we feel beauty more strongly while in heightened emotional states? Do answers to these questions depend on traits of the individual, and, in particular, on empathy?

## Aesthetics and emotion

The study of emotion in the context of aesthetics often focuses on those emotions referred to as “aesthetic emotions,” emotions like awe and the feeling of being moved (e.g., Juslin, 2013; Leder & Nadal, 2014; Menninghaus et al., 2019; Schindler et al., 2017). But surely any discrete emotion category could be involved in aesthetic experience.

Sadness has been of particular interest to music theorists and philosophers and psychologists of music (e.g., Eerola et al., 2018; Sachs et al., 2015; Vuoskoski and Eerola, 2017), in part because the observation that people tend to find sad music beautiful and pleasant seems to contradict the links between sadness and negative valence and between beauty and pleasure (Brielmann & Pelli, 2019; Kant, 1790). In response to the former (seeming) contradiction, we focus here on discrete emotion categories (e.g. sadness) rather than the valence-arousal spectrums used by Berlyne (1971) and many contemporary scholars. Rather than asking whether the perceived valence of a song predicts its beauty rating, we ask separately whether the perceived happiness and sadness levels of the song predict its beauty. *Not* casting sadness as an entirely negative emotion and *not* relying on the valence-arousal paradigm dispel the seeming paradox of the enjoyment of musical sadness.

Stimulus-focused aesthetics studies, like those about musical sadness, often investigate the relationship between “objective” stimulus features (features the experimenter ascribes to a stimulus) rather than stimulus attributes as appraised by participants (Berlyne, 1971; Silvia, 2005). In the case of emotion as a stimulus feature (e.g., a sad song), a better model of the feature’s effect on (participant-appraised) beauty might result from the feature as judged by each participant. Substituting each participant’s appraisal of perceived emotion for the single appraisal by the experimenter avoids the risk of misalignment between experimenter- and participant-appraisals. Thus, in this study we foreground the emotions that participants perceive in stimuli, rather than taking emotion qualities in stimuli as “objective.” In this paper we refer to the emotion an observer perceives as a quality of an object as “perceived emotion” (i.e. what we mean when we call a song “sad” even if it does not make us feel sad) and the emotion an observer experiences themself as “felt emotion,” since these terms are widely used for this distinction in the field. However, we acknowledge that both kinds of emotions are of course perceived by the observer.

While numerous studies have investigated perceived emotion as an input to aesthetic experience (e.g., Eerola et al., 2018; Hanich et al., 2014; Pelowski et al., 2020) and felt emotion as an output of aesthetic experience (e.g., Juslin, 2013; Leder & Nadal, 2014; Menninghaus et al., 2019), few contemporary aesthetics researchers have investigated felt emotion as an input, asking questions like “Does a person’s present emotional state affect their beauty experience” as opposed to “What emotions arise from experiences of beauty.” Taruffi and Koelsch (2014) conducted one such study, asking participants how much they like listening to happy and sad music while in a series of moods. They found that participants prefer to listen to music expressing an emotion congruent with their prior mood. Sachs et al. (2015) account for this finding by suggesting that sad music restores homeostatic affective balance to a person in distress. Both studies contribute to the growing body of research on musical sadness, and they help characterize how felt emotion affects beauty. They also highlight several questions: Is the effect of felt emotion on aesthetic response something we can measure in real time and then quantify, or does it only show up in interesting ways anecdotally and in hindsight? Are relationships between emotion and aesthetic experience consistent across object types? Are they consistent across individual traits? Bruns et al. (2023) addressed the first two questions with regard to images and music, showing that the effect of perceived and felt sadness on beauty grows with stimulus duration and is greater for music than images. In the present study we address this last question.

## Trait modulators of aesthetic experience

Empirical aesthetics research increasingly considers several trait factors, including sensitivity to aesthetic reward, openness to experience, and art expertise. For example, Trupp et al. (2023) found that sensitivity to aesthetic reward predicted the benefit of art viewing on well-being. Vuoskoski and Eerola (2011) observed that openness to experience was positively correlated with the intensity of emotional responses to music excerpts, and Silvia et al. (2015) found that openness to experience predicted the experience of awe in response to music. A series of studies have also found differences in aesthetic ratings (e.g., beauty, liking, interest) of art across participants with varying levels of art expertise (e.g., Darda & Cross, 2022; Silvia, 2013; Van Paasschen et al., 2015), and Leder et al. (2014) found variation in emotional responses to art across expertise: art experts showed attenuated emotional responses to contemporary artwork compared to non-experts.

Given the strength of these results, we measure each of these factors in the current study and discuss their effects. However, we give particular attention to one trait factor, empathy, given its especially tight theoretical and empirical connection to emotion.

## Conceptual basis of empathy

Empathy has gained interest among researchers and clinicians in recent decades, but Hall and Schwartz (2019) point out that the term has lost some conceptual coherence. In the context of interpersonal behaviors, psychopathology, and cognitive and neural processes, “empathy” takes on numerous meanings. Some of these include: the capacity to perceive others’ emotions accurately (emotion recognition), the tendency to take on another’s perspective (perspective-taking), the tendency to take on emotions of others (emotion contagion), the tendency to take on emotions in one’s environment (proximal and peripheral responsivity), and the tendency to feel care and concern for distressed others (concepts 7 and 8 in Batson, 2009). Concern for another’s welfare is the facet of empathy most closely associated with sympathy (Hall and Schwartz, 2019). Whereas sympathy is often limited to this definition, many consider empathy multidimensional, representing a combination of these and other meanings (e.g., Batson et al., 2009; Carré et al., 2013), and they often find it valuable to separate these dimensions into two categories: cognitive and affective empathy. Gladstein (1983) takes this view, arguing that empathy is a multistage process that can involve emotion contagion, identification (or recognition), and role (or perspective) taking, and he characterizes these components as cognitive or affective in nature.

To capture these aspects of empathy, we selected the Questionnaire of Cognitive and Affective Empathy (QCAE; Reniers et al., 2011) as our empathy measure. The QCAE has scales for perspective taking, online simulation, emotion contagion, proximal responsivity, and peripheral responsivity, where the first two constitute cognitive empathy and the last three constitute affective empathy.

Characterizations of empathy also vary along the trait-state divide (Cuff et al., 2016): Is empathy a relatively stable individual trait, or is it dynamic or situational? In support of the former, research indicates that empathy has stable associations with a number of other traits, like gender (Derntl et al., 2010), autism, psychopathy, and education (Thomas, Fletcher, & Lange, 1997). In support of the latter, some studies have shown that empathic responses can be situational. Empathic response seems to be stronger in people who value one another (Batson et al., 2007) and weaker in circumstances of high cognitive load (Rameson et al., 2012). This trait-state distinction did not motivate our research question or experimental design, but it does affect our interpretation of our findings.

## Aesthetics and empathy

Regardless of its precise definition, many scholars consider empathy a critical part of aesthetic experience. This link is thought to trace back to Vischer (1873), who used the German word “Einfühlung,” which means “feeling into,” to refer to a kind of perspective-taking that involves projecting oneself into an object or another body (Ganczarek et al., 2018; Jahoda, 2005). Vischer (1873) says that this “feeling into” is what facilitates profound art experiences. But Brinck (2017) criticizes this “simulation theory” of empathy and aesthetic experience, claiming that critical to empathy is not just “feeling into” the experiences of others, but also recognizing others’ experiences as distinct from one’s own. For Brinck (2017), feelings of insight, awe, and beauty arise from this recognition, not just from viewers’ direct emotional engagement with artwork, and this is core to empathy’s role in aesthetic experience.

Several recent studies lend empirical support to both theories. For example, Gernot et al. (2018) find that high emotion contagion corresponds to more emotionally congruent and more intense bodily reactions to visual art. Pelowski et al. (2018) even find that the patterns of emotion in viewers of art can match those documented in artists during the art-making process, which is presumably mediated by empathy.

Our hypothesis is that high empathy might be associated with heightened relevance of emotion to the experience of beauty. While many related studies focus on visual art, we are also interested in these effects for music. Eerola et al. (2018) provide a theoretical conjecture for the way empathy affects music listening. They suggest that components of music – like harsh or soft timbres, ascending or descending melodic phrases, and fast or slow tempos – mimic the emotionally expressive features of the human voice and thereby become emotionally expressive. Listening to emotional music is then somewhat like listening to an emotional person, meaning empathy might affect the former like it does the latter. Eerola et al. (2018) review several studies that show empathy intensifies emotional reactions that match the emotions expressed in music. Indeed, this phenomenon is captured almost explicitly in the QCAE itself, under the peripheral responsivity subscale, which includes items like “I often get deeply involved with the feelings of a character in a film, play, or novel” and “I usually stay emotionally detached when watching a film.” This theory of musical emotion from Eerola and colleagues (2018) might explain how these dimensions of empathy (as defined by the QCAE) affect emotional responses to music in addition to things like narratives and visual art where emotion is sometimes expressed more literally.

## Current study

We aim to characterize the relationship between beauty, emotion, and empathy. Unlike most related projects, we account for potential differences across four dimensions –

- locus of emotion (observer and object, i.e. felt and perceived emotions),
- discrete emotion categories (happy and sad),
- sensory modality (visual and auditory), and
- individual level of empathy

– in order to develop a more comprehensive model.

We implement the experimental paradigm from our prior work (Bruns et al., 2023), asking participants to take a mood questionnaire and then rate a set of songs and images in terms of perceived happiness, sadness, and beauty. We include a mood intervention and additional mood questionnaire after the first half of stimulus trials to broaden the range of participant moods at play during the survey. And we include the Questionnaire for Cognitive and Affective Empathy (QCAE) so that we can assess the effect of empathy on the relationship between emotion and beauty.

Bruns et al. (2023) note that much of the literature on emotion and aesthetic response attempts a general explanation for *why* people enjoy stimuli like sad music. They then presented data that quantified to *what extent* people in fact like sad music, finding that more emotional (sad and happy) music is judged more beautiful than less emotional music, while happy (but not sad) images are judged more beautiful than less happy ones. The present study continues this work, testing the hypothesis that empathy might amplify these effects.

## Methods

The present study replicates the methods from Bruns et al. (2023) on a new sample, with several measures added: the Aesthetic Fluency Scale (Cotter et al., 2023), the Openness to Experience scale within the Big 5 (McCrae & Greenberg, 2014), and impulsiveness (Cyders et al., 2014). We update Bruns and colleagues’ method of analysis of modulating factors like the Questionnaire for Cognitive and Affective Empathy (QCAE; Reniers et al., 2011), and we apply this method to other modulating factors in order to gauge their relative effects.

### Participants:

170 participants recruited through Prolific Academic (https://prolific.co/) took part in the experiment. 164 were included in the analysis, with data from 6 excluded because they failed the attention checks described in the “Procedure” section. Of the 164, 79 identified as men, 77 as women, and 8 as non-binary. 65 participants identified as White, 37 as Asian, 19 as Black, 17 as Hispanic, and 26 as mixed race or “other” race/ethnicity. Their ages ranged from 18 to 28 years (M = 24.0, SD = 2.7). We limited the participant age range because we needed the stimulus set to be relevant to as much as the group as possible (i.e., show variation in beauty ratings, including high ratings), and music preferences vary widely across age groups. All participants spoke English as their first language, were U.S. nationals, had no hearing impairments, and had normal or corrected-to-normal vision. In terms of highest level of education, 37 participants had completed high school at the time of the study, 49 had completed some college, 15 had an Associate’s degree, 52 had a Bachelor’s degree, 8 had a Master’s degree, and 3 had a Ph.D. On average, participants had 2.1 years of visual art training (SD = 3.7) and 3.1 years of music training (SD = 4.4). 34 participants had 4 or more years of visual art training and 49 had 4 or more years of music training. All participants gave informed consent in accordance with the declaration of Helsinki. This experiment was approved by the New York University Committee on Activities Involving Human Subjects (UCAIHS; IRB-FY2020–4100).

#### Stimuli

We used the same set of 12 visual art images, 12 nature photographs, and 24 20-second song excerpts that Bruns et al. (2023) used. They give a detailed description of the search and selection process for these stimuli. We will highlight just the selection criteria here.

Based on pilot data, all stimuli were selected to be judged as very beautiful to at least some participants so that as many participants as possible would experience intense beauty in response to some of the stimuli. Each stimulus type (art images, nature photos, music) includes a balanced proportion of stimuli that participants would consider very happy, very sad, or neither. All stimuli were unfamiliar to most pilot participants to avoid familiarity introducing a confound. Because the survey design involves two blocks of 24 trials each, one before and one after a mood induction phase, stimuli were selected in pairs, where the elements in each pair had comparable beauty, happiness, and sadness ratings, and where image pairs contained images of the same orientation (portrait or landscape) and with the same subject matter. Elements of the pairs were pseudo-randomly assigned to each block, and blocks were counterbalanced. This helps avoid any consequential differences in our main measures before and after mood induction caused by stimulus assignment.

We also note that neither the image nor the song sets were selected to be representative of all genres and geographies. The visual artwork is Western and representational, and all music is pop (defined broadly), to help eliminate geography and genre as a confound. We did not want within-participant beauty rating variance to come from genre; we wanted it to come from the emotion factors of interest to us.

**Figure 1:**
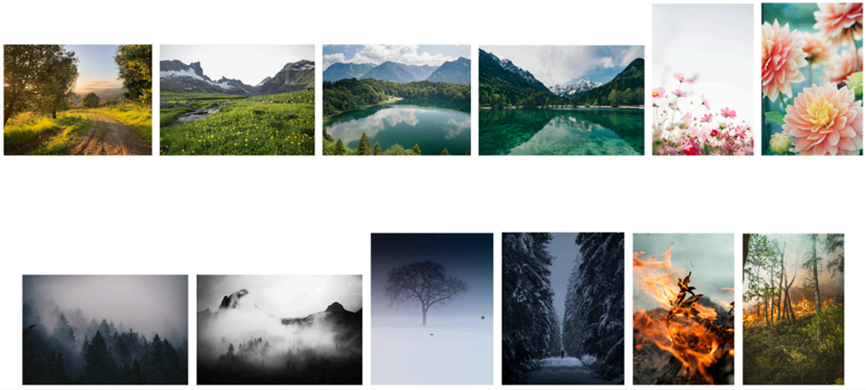
All 12 nature photograph stimuli taken from Unsplash (https://unsplash.com/), sorted approximately by mean happiness rating (from pilot data) in descending order. The order is adjusted so that image pairs are side by side.

All stimuli and summary statistics of their ratings can be found here: URL. Nature photographs are shown in Fig. 1.

#### Mood induction videos

We presented a mood induction video halfway through the survey to increase participants’ happiness or sadness level or to leave their mood unchanged (control). A primary goal of this study is to understand the possible causal (not just correlational) relationship between felt emotion and beauty. To speak to cause, we did an intervention. This intervention ensured that the majority of participants rated stimuli while in two different emotional states, meaning that the effects of felt emotion we report are not merely effects of individual difference in disposition or another confound but are indeed effects of felt emotion. The intervention also broadened the range of participant moods.

We used the same videos Bruns et al. (2023) used, which they showed were effective:

- Happiness induction: Mixtape Medley with Ariana Grande and Kelly Clarkson (https://www.youtube.com/watch?v=zJUXTrKRdf4&t=1s)
- Sadness induction: Jack’s death scene from *Titanic* (1997) (https://www.youtube.com/watch?v=w6OzanamcI8)
- Neutral mood induction (control): Clip from the film *Blue* (1993) during which the character Olivier shifts papers on a desk, sourced from the film clip database put together by Schaefer et al. (2010)

The survey randomly assigned participants either the happy, sad, or neutral mood induction video. 55 participants were shown the happy mood induction video, 54 were shown the sad mood induction video, and 56 were shown the neutral mood induction video.

#### Measures

Stimulus trial questions are listed below.

1. How much do you **like** this image/song? (*7-pt. scale from “Not at all” to “A lot”*)
2. How much **beauty** do you feel from this image/song right now? (*7-pt. scale from “None at all” to “A lot”*)
3. How much **happiness** does this image/song evoke? (*7-pt. scale from “None at all” to “A lot*”)
4. How much **sadness** does this image/song evoke? (*7-pt. scale from “None at all” to “A lot”*)
5. How **familiar** are you with this particular image/song? (*7-pt. scale from “Not at all” to “Very familiar”*)
6. How **familiar** are you with images/songs like this one? (*7-pt. scale from “Not at all” to “Very familiar”*)

We used the Positive and Negative Affect Score (PANAS) mood questionnaire (Watson, 1998) to collect data about participants’ present mood. To the PANAS questionnaire we appended two additional emotion items—“happy” and “sad”—so that we could leverage both the PANAS positive and negative affect scores as well as simple measures for happiness and sadness, since the questions we asked about the emotion evoked by stimuli asked about happiness and sadness. The questionnaire asked participants to “Indicate to what extent you feel this way right now, that is, at the present moment,” and all emotion items used a 5-point Likert scale from “Very slightly or not at all” to “Extremely.”

We also collected the following demographic and trait measures from each participant: age, gender, highest level of education, race/ethnicity, visual art and music training questions, the Aesthetic Responsiveness Assessment (AReA; Schlotz et al., 2021), the Barcelona Music Reward Questionnaire (BMRQ; Mas-Herrero et al., 2013), the Questionnaire of Cognitive and Affective Empathy (QCAE; Reniers et al., 2011), the Aesthetic Fluency Scale (Cotter et al., 2023), the Openness to Experience scale within the Big 5 (McCrae & Greenberg, 2014), and impulsiveness (Cyders et al., 2014).

### Survey:

We programmed the survey on Qualtrics (https://www.qualtrics.com/). We instructed participants to use a desktop computer or laptop to complete the study, not a smartphone or tablet, but we did not verify compliance.

#### Procedure

**Figure 2:**
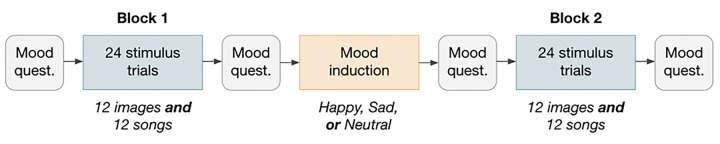
Survey structure. Participants were randomly assigned either a happy, sad, or neutral mood induction video. In Block 1 and 2 of stimulus trials, the order of presentation of each stimulus type was randomized (either all images or all songs first). The mood questionnaire used was the PANAS (Watson, 1998).

After giving consent and reading brief instructions about the contents, participants rated the 24 songs, 12 art images, and 12 nature photographs and took the PANAS mood questionnaire following the structure shown in Fig. 2. Participants rated all the image stimuli or all the song stimuli first within each block with the order of stimulus type presentation counterbalanced and the presentation order of each individual stimulus randomized. Trait and demographic questions were presented at the end of the survey.

We included two attention checks. One was in the form of an additional auditory stimulus trial, which included a voice recording in place of a song clip with the same stimulus trial questions listed for all other auditory stimuli. The recording instructed participants to mark the rightmost answer choice for every question on the page. This attention check also ensured participants’ audio was functional. Additionally, at the end of the survey, we asked participants how attentive they had been throughout the survey on a 7-point scale from “Not at all attentive” to “Very attentive.”

### Analysis:

We conducted all analyses using R (Version 4.2.0) in RStudio. Of our total 170 participants, we excluded the three who failed the audio attention check, the two who gave self-reported attentiveness ratings below 4 (“Somewhat attentive”) on the 7-point scale, and one who gave a “1” rating for every question in the survey. After these exclusions, attentiveness was self-rated at 5.7 ± 0.6 (M ± SD). We also excluded 70 individual trials (0.9% of the 164 participants × 48 stimuli = 7872 total trials) with trial duration beyond the following threshold: Q3 + (9 × IQR) = 164 sec. These trials well-exceeded the median trial duration of 19.4 sec. 36 of the 164 participants had at least one trial with a duration exceeding the 164-sec threshold, and individual participants had at most four such trials. Bruns et al. (2023) showed that stimulus duration affects the relationship between beauty and emotion for music and images, so we account for duration in our present models. We removed these outlier trials because they pose risk to model fit. After these trial exclusions, the median trial duration was 19.2 sec and mean was 22.6 sec.

After these participant and trial exclusions, we re-scaled all Likert variables (e.g. beauty, PANAS, QCAE, etc.) to 10-point scales to make results more interpretable, we mean-centered all variables, and we arranged our dataset so that each participant’s stimulus ratings were associated with their responses to the mood questionnaire they took prior to seeing that stimulus block. All questions in the survey were required, so we had no missing data.

Next, we tested whether the mood induction was successful. We performed two-tailed paired t-tests on the happiness and sadness ratings from the mood questionnaires participants completed before and after viewing the mood induction video.

**Figure 3:**
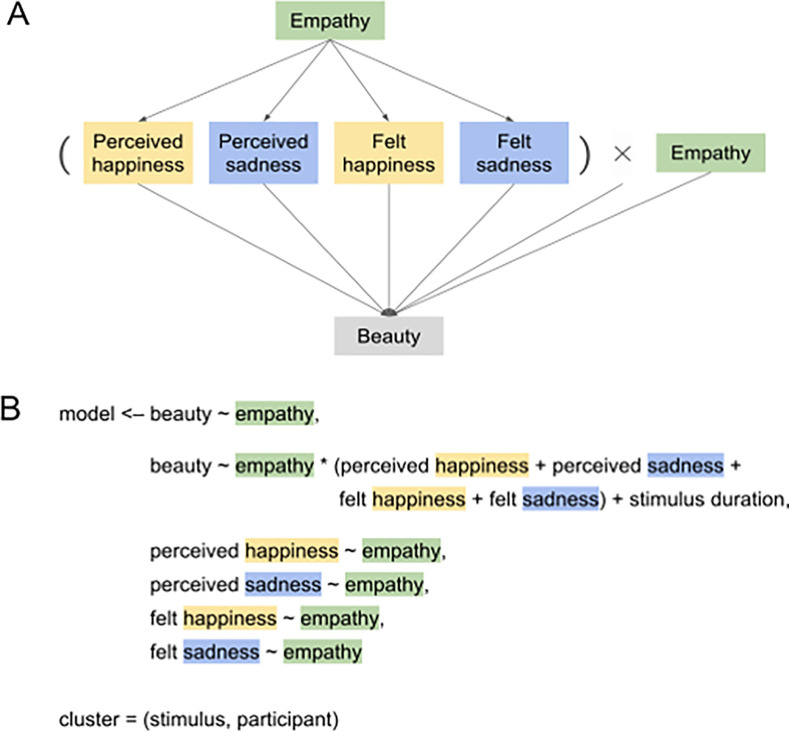
Structural equation model (SEM) structure. (A) Simplified diagram of model structure. (B) R pseudocode, with “~” representing “regressed on,” “*” representing an interaction, and “+” joining predictors in a multiple regression. The model assesses the effect of empathy on the relationship between four emotion factors and beauty judgments of images and songs, accounting for direct effects of trait empathy on emotion and beauty. The model accounts for stimulus duration and for random variation in measures at the participant and stimulus level (using the “cluster” parameter in *lavaan*’s “sem” function). Perceived emotion and beauty correspond to Likert ratings for each stimulus presentation, and felt emotion corresponds to the happiness and sadness simple ratings that each participant provided in the mood questionnaire before each stimulus trial block. “Empathy” corresponds to each participant’s total score on the Questionnaire of Cognitive and Affective Empathy (QCAE; Reniers et al., 2011).

Then we fit structural equation models (SEMs) to our data using the *lavaan* package (Rosseel, 2012) in R. We ran two separate models, one for images and one for music. Each one included the following three paths: beauty regressed on empathy; felt and perceived happiness and sadness regressed on empathy; and beauty regressed on the interaction between empathy and felt and perceived happiness and sadness, accounting for trial duration (see pseudocode in Fig. 3B). In these models, perceived emotion corresponds to the Likert rating after each presentation of a particular stimulus, and felt emotion corresponds to the happiness and sadness ratings that each participant provided in the mood questionnaire before each block. (We used these single mood questionnaire emotion ratings rather than the PANAS positive and negative affect scores so that felt and perceived emotion measures are analogous.) Empathy and these four emotion factors are not correlated, and their simple relationships with beauty are approximately linear (satisfying assumptions of the model). This model structure treats empathy as exogenous and the emotion and beauty measures as endogenous. In this way, we assess our target measure – the role of empathy in the relationship between emotion and beauty – while accounting for and measuring direct effects of empathy on emotion and beauty variables. We also accounted for random variation at the participant and stimulus level by including these two factors as clusters in the model. The SEM structure is visualized in Fig. 3.

We factor the mood induction into the analysis using the participant’s self-reported mood ratings rather than the mere fact that we showed them a video intended to induce mood. This measurement method avoids the presumption that the mood induction had the same effect for each individual.

We conducted a power analysis using the “SEM based on RMSEA” tool in WebPower (Zhang & Yuan, 2018). With our sample size of 164, degrees of freedom 40, RMSEA (root mean square error of approximation) for the null hypothesis set to 0.05 and for the alternative hypothesis set to 0.09, and significance level 0.05, our SEM model results have a power value of 0.82.

## Results

### Effect of empathy on the relationship between emotion and beauty of music.

We find direct effects of perceived happiness, perceived sadness, and trait empathy on song beauty. Accounting for other relationships in the model, and with empathy at sample-mean level, an extra point of song happiness predicts an extra 0.46 points of beauty, and an extra point of song sadness predicts an extra 0.51 points of beauty (all measures on a 10-point scale). And with emotion ratings at sample-mean level, an extra point of empathy predicts an extra 0.37 points of beauty. Felt emotion factors have no significant effect on song beauty.

Empathy also predicts increases in each emotion measure. Accounting for other relationships in the model, an extra point of trait empathy predicts an extra 0.26 points of perceived song happiness, 0.27 points of perceived song sadness, 0.18 points of felt happiness, and 0.15 points of felt sadness.

**Figure 4:**
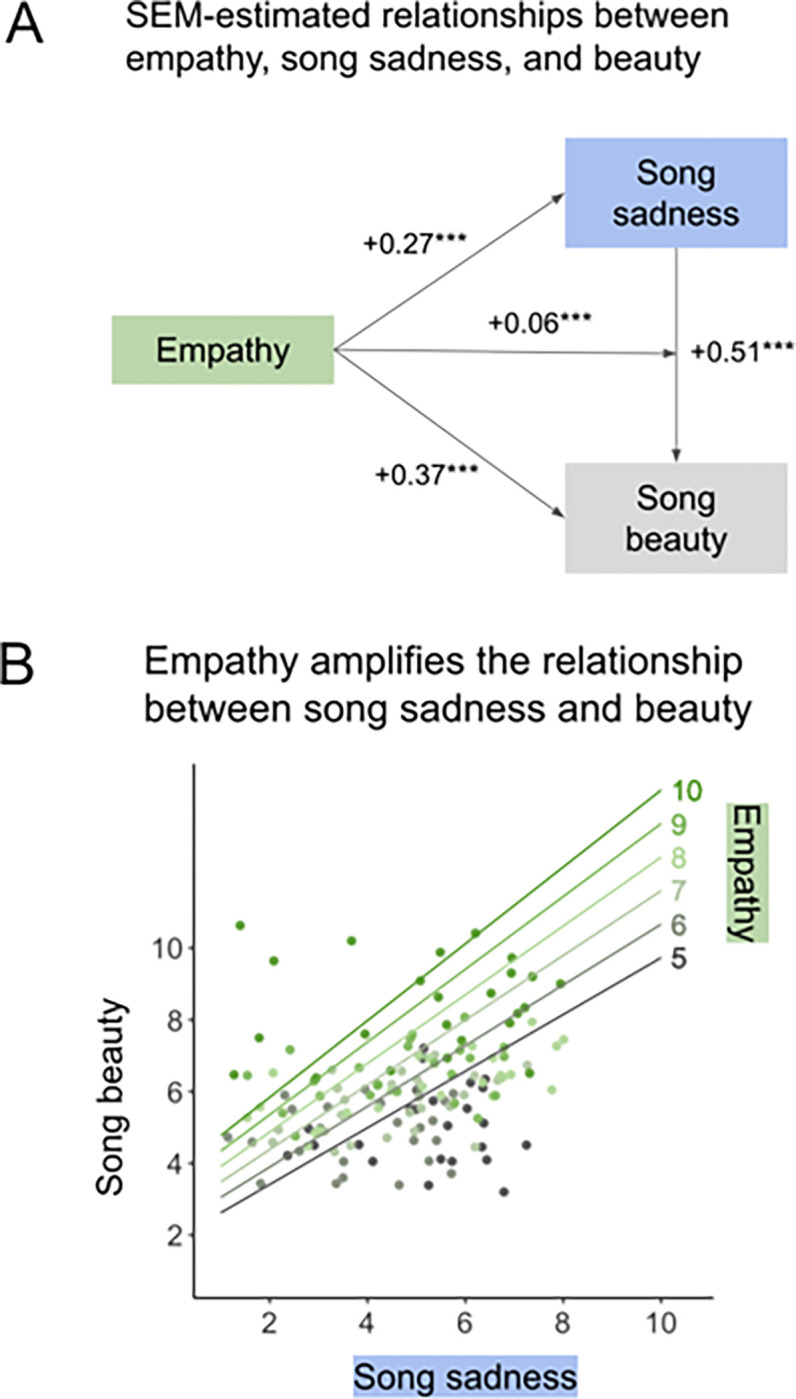
(A) Portion of the Table 1 structural equation model (SEM) that is most relevant to the literature on musical sadness. The +0.27 and +0.37 coefficients represent the direct effects of empathy on song sadness and song beauty, accounting for all other factors in the model (see Figure 3 for model schematic). The +0.51 coefficient represents the partial effect of song sadness on song beauty, when trait empathy is at the sample mean. The +0.06 coefficient represents the effect of the interaction between empathy and song sadness on song beauty: on average, each 1-point increase in trait empathy predicts a 0.06-point increase in the magnitude of the relationship between song sadness and beauty. * *p* < 0.05, ** *p* < 0.01, *** *p* < 0.001. (B) Song sadness × song beauty trendlines at each empathy level represented in our sample (sample QCAE scores range from 4.1 to 9.6 with mean 7.5 and SD 0.94). Datapoints represent the mean sadness and beauty ratings for each song stimulus for participants at each empathy level. All measures were rescaled to a 10-point Likert scale.

Most critically, we find that higher trait empathy is associated with stronger relationships between emotion and beauty. All else equal, each extra point of trait empathy amplifies the relationships between song happiness and beauty by 0.04 points, between song sadness and beauty by 0.06 points, and between felt happiness and beauty by 0.06 points, on average. We visualize the structure of one of these interactions in Figure 4. Figure 4 shows the magnitudes of the relationship between trait empathy, song sadness, and song beauty. All of these relationships are highly significant (*p* < 0.001).

Table 1 outlines these results, which come from the structural equation model assessing the following relationships for music stimuli: the effect of empathy on beauty ratings, the effect of empathy on the four emotion measures, and the effect of the interaction between empathy and emotion on beauty. The model accounts for stimulus duration and for random variation in measures at the participant and stimulus level. The structure of these relationships is visualized in Figure 3.

### Effect of empathy on the relationship between emotion and beauty of images.

We find direct effects of trait empathy and all four emotion variables on image beauty (see the first five lines in Table 2, which shows results for the same structural equation model as in Table 1, but this time for images rather than music). Accounting for other relationships in the model, and with emotion ratings at sample-mean levels, an extra empathy point predicts an average 0.27-point beauty increase. And with trait empathy at the sample-mean level, increases in perceived emotion factors predict increased beauty – a 0.58-point beauty increase per image happiness point and a 0.12-point beauty increase per image sadness point, on average – while increases in felt emotion factors predict slight decreases in beauty – a 0.06-point beauty decrease per felt happiness point and a 0.03-point beauty decrease per felt sadness point (all *p* < 0.01).

Like in the music model, empathy again has positive direct relationships with emotion measures. Accounting for other relationships in the model, an extra trait empathy point predicts average increases of 0.26 points in perceived image happiness, 0.19 points in felt happiness, and 0.15 points in felt sadness.

In terms of the interaction between empathy and emotion, for images, higher trait empathy is associated with stronger relationships between felt but not perceived emotion and beauty. All else equal, a one-point increase in trait empathy amplifies the relationships between both felt happiness and beauty and felt sadness and beauty, each by 0.06 points, on average.

**Figure 5:**
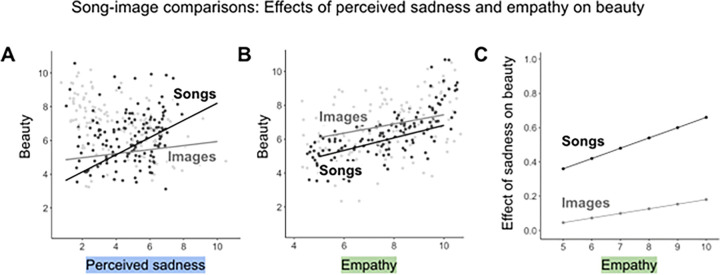
(A) and (B) Trendlines use slopes and intercepts from SEMs for songs and for images, showing positive simple relationships between perceived sadness and beauty and between empathy and beauty. Datapoints represent mean sadness and beauty ratings for each stimulus for participants at each empathy level. All measures are on a 10-point Likert scale. (C) Lines represent the effect of the interaction between perceived sadness and empathy on beauty for songs (*p*< 0.001) and images (*p* > 0.05).

Unlike the music model, the image model does not show significant interactions between empathy and perceived emotion. Figure 5 visualizes the difference in the relationship between perceived sadness, empathy, and beauty for images vs. music. Panel A shows that song sadness predicts a substantial increase in song beauty (*β* = 0.51, *p* < 0.001), while image sadness only predicts a marginal increase in image beauty (*β* = 0.12, *p* = 0.002). Panel B shows comparable increases in song and image beauty associated with empathy. Finally, Panel C shows the stronger interaction between empathy and perceived sadness for songs than images.

### Distributions and correlations of measures in SEMs.

We verified that the distribution of each stimulus measure (beauty, happiness, and sadness) aligned with our stimulus selection criteria (see Supplementary Materials), and we compared our sample empathy (QCAE) score distribution to the one Reniers et al. (2011) report in their paper introducing the QCAE. Our sample empathy statistics are 92.9±11.18 (*M*±SD), and Reniers (2011) reports statistics of approximately 92.3±12.2 on a sample of 925 individuals (using the original QCAE scale, before we rescaled to a 10-point scale for interpretability).

**Figure 6:**
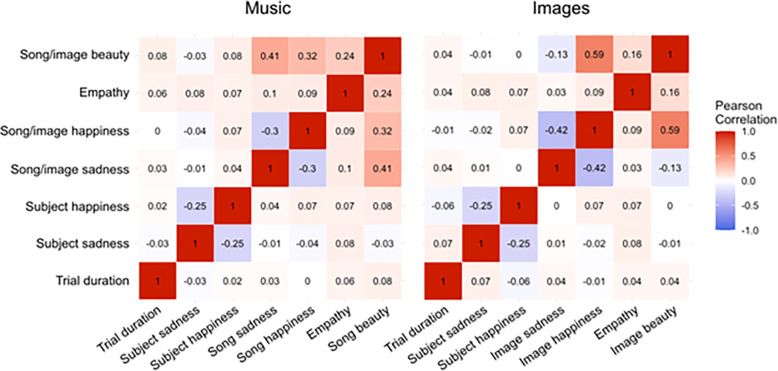
Pearson correlation heatmaps for variables in the song (left) and image (right) SEMs.

Figure 6 shows Pearson correlations between all measures included in SEMs for music and images. These show that all predictors in the SEMs (the emotion variables, empathy, and trial duration) have correlations of at most 0.1, confirming multicollinearity is not at play in the models. They also show moderate negative correlations between happiness and sadness emotion factors, as we would expect, as well as moderate positive correlations between song emotion and beauty and between image happiness and beauty.

### Comparing other traits with empathy as a predictor of beauty and emotion.

In the Supplementary Materials we report analogues to Tables 1 and 2 for other potential modulators of the relationship between emotion and beauty. We include structural equation model results with aesthetic reward, music reward, art fluency, openness to experience, and impulsiveness swapped in for empathy. Results are comparable but generally lower magnitude compared to those for empathy. So why is empathy a better predictor in this study? We infer that its link to emotion, and emotion’s link to beauty, make it play an especially important role in the relationship between beauty and emotion.

**Figure 7:**
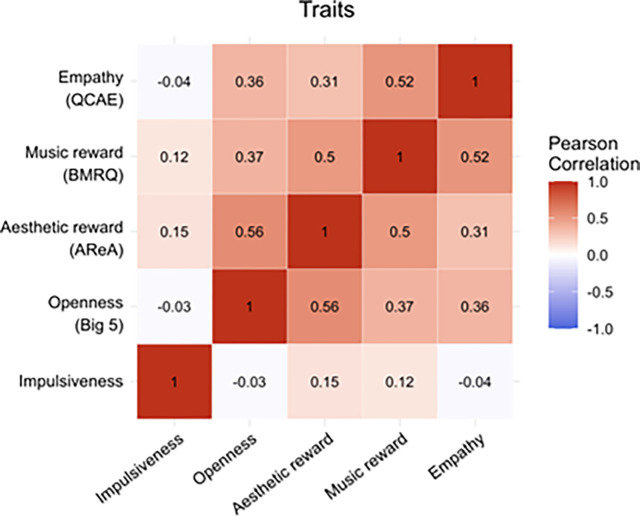
Pearson correlation heatmap for trait variables we hypothesized might modulate the relationship between emotion and beauty.

Table 3 shows results from 48 structural equation models, each assessing the simple effects of one of the trait variables on one of the stimulus variables (beauty, perceived happiness, perceived sadness) or state variables (felt happiness, felt sadness). We ran these as separate models since several of the traits are correlated (see Figure 7). We find that empathy is generally the strongest predictor of the response variables. Aesthetic reward and music reward, however, are comparable predictors, which helps justify their use in other empirical aesthetics and music perception studies.

### Mood manipulation check.

We compared participants’ responses to mood questionnaires before and after viewing mood induction videos to gauge effectiveness of the mood induction during the main study. Tables 4–6 contain results from two-tailed paired t-tests performed on participants’ ratings to the “Happy” and “Sad” items appended to the PANAS mood questionnaire. Results show that the happiness and sadness induction videos both had the desired effect on participants’ happiness and sadness ratings. After the happiness induction, “Happy” ratings showed a nearly significant 0.58-point increase (*p* = 0.058) out of 10, and “Sad” ratings showed a 0.95-point decline (*p* < 0.001). After the sadness induction, “Happy” ratings showed a 1.48-point decline (*p* < 0.001) out of 10, and “Sad” ratings showed a 1.85-point increase (*p* < 0.001). The neutral mood induction led to no significant change in happiness or sadness ratings, as expected. We also find that, after the mood induction, the mean happiness rating was significantly higher for participants in the happiness induction group compared to the sadness and neutral induction groups, and the mean sadness rating was significantly higher for participants in the sadness induction group compared to the happiness and neutral induction groups (see Supplementary Materials).

## Discussion

This study investigates how empathy affects the link between beauty and emotion. We account for potential differences across several dimensions: locus of emotion (observer and object, i.e. felt and perceived emotions), emotional valence (happy and sad), and sensory modality (visual and auditory). 164 18- to 28-year-old U.S.-based participants rated 24 images and 24 songs in terms of perceived beauty, happiness, and sadness, and all participants took the PANAS mood questionnaire several times throughout the survey (see Fig. 2) as well as a series of trait questionnaires, including the Questionnaire of Cognitive and Affective Empathy (QCAE) (Reniers et al., 2011). Halfway through the survey, participants viewed a mood induction video intended to make them happier or sadder or to leave their mood unchanged. We ran structural equation models to assess direct effects of empathy on beauty and emotion measures (felt and perceived happiness and sadness) in addition to the effect of interactions between empathy and emotion on beauty, accounting for stimulus duration. As we hypothesized, results show that relationships between emotion and beauty are amplified by trait empathy.

### Empathy affects the relationship between emotion and beauty

We find that more emotional songs (songs with higher happiness and sadness ratings) are judged more beautiful, especially for participants high in empathy. The effect of perceived emotion on song beauty for participants near the maximum sample empathy level is nearly double the size of that for participants near the minimum. We might understand the result that empathy amplifies the beauty of happy music as simply following directly from one conceptual facet of empathy: the tendency to take on emotions in one’s environment (proximal and peripheral responsivity). Beauty and happiness are both strongly associated with pleasure (Brielmann & Pelli, 2019; Kant, 1790), so it seems intuitive that people find happy songs especially beautiful, and that empathy would strengthen this effect.

The result that empathy amplifies the beauty of sad music, however, is a bit less intuitive, though it aligns with comparable studies on musical sadness and empathy. Vuoskoski and Thompson (2012), for example, show that the enjoyment of musical sadness is associated with high empathic concern and low personal distress, as measured by the Interpersonal Reactivity Index (IRI; Davis, 1980). Huron and Vuoskoski (2020) claim that feelings of compassion might underlie this effect. Because compassion is closely related to empathic concern and is a pleasurable prosocial emotion, Huron and Vuoskoski (2020) suggest the pleasure of sad music might come in part from the pleasure of compassion. Our findings about sad music are compatible with Vuoskoski and Thompson’s (2012) result and with Huron and Vuoskoski’s (2020) theory.

Results for images are slightly different. We replicate the Bruns and colleagues (2023) result that perceived happiness but not sadness predicts increased image beauty, affirming the higher share of psychology literature that musical sadness occupies relative to image sadness. And unlike for music, empathy does not strengthen this perceived happiness effect. These results seem not to align with Gernot and colleagues’ (2018), but we can intuit why. They found that participants high in emotion contagion gave higher liking ratings to high-valence visual art and lower liking ratings to low-valence visual art than participants lower in emotion contagion did. But we find that participants higher in empathy give higher beauty ratings to songs that they judge as happy and to songs they judge sad, and we find no significant interaction between empathy and perceived emotion in images. We postulate two of many possible reasons for this supposed discrepancy. First, their stimuli were classified in terms of valence (pleasantness level) rather than discrete emotions, and sadness need not always be unpleasant. Second, we used different methods of analysis. Our models account for direct effects of empathy on emotion and beauty ratings, and they account for random variation at the stimulus and participant level. These factors may explain the difference between our results and those of Gernot and colleagues’ (2018). These differences in methodology suggest our findings and theirs might be taken as complementary rather than contradictory.

In addition to the interactions between empathy and perceived emotion (stimulus ratings) that we find in the context of music, we find significant interactions between empathy and felt emotion (mood questionnaire ratings) in the context of images and music. Our results indicate that more empathic participants judge images as more beautiful when they feel especially happy or sad, and more empathic participants judge songs as more beautiful when they feel especially happy. These results account for direct effects of empathy on felt happiness and sadness and on beauty, meaning the result cannot be explained by these direct effects alone. The idea that the felt emotions of empathic people might strengthen their experience of beauty is reminiscent of Vischer’s (1873), Lipps’ (1903), and Brinck’s (2017) theories of empathy and aesthetic experience. If one is especially prone to “feeling into” art objects, then they might be more likely to be moved by and ultimately find beauty in those objects when they are emotional.

### Empathy predicts direct increases in beauty and in felt and perceived emotions

In addition to its effect on beauty through interactions with emotion, we find that empathy is positively associated with the beauty of both music and images. This result further affirms the theorized importance of empathy to aesthetic experience. We also find that empathy predicts emotion ratings directly. More empathic participants gave higher ratings of song happiness and sadness, image happiness, and felt happiness and sadness, on average. Because we did not establish some ground truth happiness and sadness level for each stimulus (based on artist intent, the judgment of experts, or the judgment of a large sample), we cannot verify whether empathy was associated with more accurate emotion recognition, as other studies have investigated (e.g., Besel & Yuille, 2010; Wöllner, 2012). However, these direct effects suggest a simple positive association between empathy and the magnitude of emotions one might perceive and experience amid emotional objects, an idea again compatible with Vischer’s (1873), Lipps’ (1903), and Brinck’s (2017) theories.

### Are other traits as relevant to beauty and emotion as empathy is?

We compared empathy with the following traits: aesthetic reward (AReA), music reward (BMRQ), art fluency (AFS), openness to experience (Big Five), and impulsiveness. We note offhand that several of these measures exhibit moderate or strong positive correlations with each other, including empathy and music reward, aesthetic reward and openness, and empathy and openness (see Fig. 6). Huron and Vuoskoski (2020) point out these associations as potential challenges in determining which traits best predict emotion and aesthetic response, and several studies have worked to disentangle them (e.g., Sattmann & Parncutt, 2018). To avoid risk of multicollinearity, we ran structural equation models with only one of these traits included as a predictor at a time and then compared beta values by computing z-scores and p-values using these betas and their standard errors.

We find that empathy is not only the strongest predictor of emotion’s positive link to beauty (see Supplementary Materials); empathy is also the strongest direct predictor of song beauty and among the strongest predictors of image beauty, with statistically equivalent effect sizes to aesthetic reward and art fluency (see Table 3).

That said, we can identify several compelling effects in the models of beauty and emotion with these other traits in place of empathy (see Supplementary Materials). For example, unlike for empathy, high art fluency and high aesthetic reward are associated with dampened relationships between perceived emotion and image beauty, suggesting that, while on average more emotional images might be judged more beautiful, this effect is weak for participants with more art experience. Winston and Cupchik’s (1992) work might explain this. They find that less experienced art viewers are more likely to invoke their subjective emotional responses to explain their art preferences, while more experienced viewers are more likely to invoke formal qualities of the artwork. Thus, while empathy remains the focus of this paper, our experimental paradigm also unveils compelling effects for other traits.

### What does this suggest about the nature of empathy and aesthetic experience?

The aesthetic triad model says aesthetic experiences consist of sensory-motor, emotion-valuation, and knowledge-meaning facets (Chatterjee & Vartanian, 2014). Our results suggest that empathy gives added weight to the emotion-valuation facet, while art fluency might give it less relative weight (in favor, perhaps, of the knowledge-meaning facet). This effect does, of course, depend on the kind of object(s) involved in the experience. While empathy might augment listeners’ enjoyment of musical sadness, our results suggest it does not augment viewers’ enjoyment of sadness perceived in images. de Wied et al. (1995) find an effect for film that is comparable to our effect for music: high-empathy participants felt both more distress and more enjoyment in response to cinematic tragedy than lower-empathy participants did. Studies by Hanich et al. (2014) and Vuoskoski and Eerola (2017) suggest why this might be so. They show that the enjoyment of sadness in film (Hanich et al., 2014) and music (Vuoskoski & Eerola, 2017) is mediated by feelings of being moved. Though not yet confirmed, it is possible that our results might be explained in part by differences across music vs. images in feelings of being moved. If participants are not moved by the images in the context of our online experiment, and if feelings of being moved are required for the enjoyment of sadness in images, as they appear to be for film and music, then we might not expect to see a strong association between image sadness and beauty.

So far we have focused on the effect empathy has on aesthetic experience, rather than the effect aesthetic experience might have on empathy. But Sherman and Morrissey (2017) discuss this latter effect, reviewing literature on the way art and aesthetic experience cultivate other-understanding. In doing so, they show that art appreciation engages processes involved in social interaction, such as cognitive perspective-taking and emotion recognition and contagion. Zhang et al. (2014) conducted one such study, finding that exposure to beautiful nature was associated with heightened prosociality. Together, our work and theirs show that empathy, emotion, and beauty are interrelated, likely amplifying one another. They make the case for the socio-epistemic value of both art and empathy – in particular its cognitive perspective-taking or other-understanding dimension. We look more closely at empathy’s emotional dimension. Our findings suggest that empathy gives us a greater capacity to find beauty in the midst of emotion.

## Conclusion

We conclude that empathy boosts the effect of emotion on beauty. We used structural equation models to assess how empathy and emotion interact to affect beauty, accounting for direct effects of empathy on beauty and emotion. We assessed four emotion variables: participants’ felt happiness and sadness (mood questionnaire ratings) and perceived happiness and sadness (stimulus ratings). Results show that more emotional songs are judged more beautiful, especially for participants high in empathy. For images, however, perceived happiness is much more predictive of image beauty than sadness is, and this effect is not amplified by empathy, as it is for music. Yet, empathy predicts increased beauty for both songs and images, and only highly empathic participants judge both songs and images as more beautiful when they feel more emotional. Vischer (1873) and Lipps (1903) were the first to claim that empathy deepens or may even be required for aesthetic experience, and many contemporary scholars agree with them (Brinck, 2017; Eerola et al., 2018). Our results strengthen this claim. And rather than focusing on just one facet of aesthetic experience, we systematically examine the effect of empathy across the dimensions of image vs. music, happy vs. sad, and felt vs. perceived emotion. This allowed us to discover important differences in the beauty of images and music. In summary, we find that:

1. Felt and perceived emotions produce more beauty.
2. This is more so in more empathic people.
3. This is more so with music than with images.

## Figures and Tables

**Table 1.** Structural equation model (SEM) result for songs (*N* = 164).

Response variable	Predictor variable	Estimate	SE	z	p	
**beauty**	**empathy**	**0.37**	**0.03**	**13.01**	**< 0.001**	***
**beauty**	**perceived happiness**	**0.46**	**0.02**	**21.23**	**< 0.001**	***
**beauty**	**perceived sadness**	**0.51**	**0.02**	**23.28**	**< 0.001**	***
beauty	felt happiness	0.00	0.01	−0.06	0.956	
beauty	felt sadness	−0.01	0.01	−0.83	0.407	
**beauty**	**stimulus duration**	**0.01**	**0.00**	**3.04**	**0.002**	**
**beauty**	empathy * perceived happiness	**0.04**	**0.01**	**4.62**	**< 0.001**	***
**beauty**	empathy * perceived sadness	**0.06**	**0.01**	**5.11**	**< 0.001**	***
**beauty**	empathy * felt happiness	**0.06**	**0.01**	**4.83**	**< 0.001**	***
beauty	empathy * felt sadness	0.00	0.02	0.20	0.845	
**perceived happiness**	**empathy**	**0.26**	**0.05**	**5.06**	**< 0.001**	***
**perceived sadness**	**empathy**	**0.27**	**0.05**	**5.62**	**< 0.001**	***
**felt happiness**	**empathy**	**0.18**	**0.01**	**26.49**	**< 0.001**	***
**felt sadness**	**empathy**	**0.15**	**0.01**	**13.48**	**< 0.001**	***

The model explains 49% of song beauty variance. Bold values indicate statistical significance. Interaction factors are highlighted in gray. All measures are mean-centered on a 10-point Likert scale.

* *p* < 0.05

** *p* < 0.01

*** *p* < 0.001.

**Table 2.** Structural equation model result for images (N = 164).

Response variable	Predictor variable	Estimate	SE	z	p	
**beauty**	**empathy**	**0.27**	**0.03**	**8.43**	**< 0.001**	***
**beauty**	**perceived happiness**	**0.58**	**0.04**	**15.72**	**< 0.001**	***
**beauty**	**perceived sadness**	**0.12**	**0.04**	**3.12**	**0.002**	**
**beauty**	**felt happiness**	**−0.06**	**0.02**	**−3.52**	**< 0.001**	***
**beauty**	**felt sadness**	**−0.03**	**0.01**	**−2.69**	**0.007**	**
**beauty**	**stimulus duration**	**0.01**	**0.00**	**2.20**	**0.028**	*
beauty	empathy * perceived happiness	−0.01	0.01	−0.92	0.355	
beauty	empathy * perceived sadness	0.01	0.02	0.55	0.584	
**beauty**	empathy * felt happiness	**0.06**	**0.01**	**5.05**	**< 0.001**	***
**beauty**	empathy * felt sadness	**0.06**	**0.02**	**2.83**	**0.005**	**
**perceived happiness**	**empathy**	**0.26**	**0.06**	**4.33**	**< 0.001**	***
perceived sadness	empathy	0.10	0.07	1.51	0.132	
**felt happiness**	**empathy**	**0.19**	**0.01**	**23.58**	**< 0.001**	***
**felt sadness**	**empathy**	**0.15**	**0.02**	**8.63**	**< 0.001**	***

The model explains 43% of image beauty variance. Bold values indicate statistical significance. Interaction factors are highlighted in gray.

* *p* < 0.05

** *p* < 0.01

*** *p* < 0.001.

**Table 3.** Beta values from 48 structural equation models with response variable designated by each column label, predictor variable designated by each row label, and random intercepts for both participant and stimulus.

		Response variable															
	
		*Song*						*Image*						*Felt*			
	
		*Beauty*		*Happiness*		*Sadness*		*Beauty*		*Happiness*		*Sadness*		*Happiness*		*Sadness*	
	
**Predictor variable**	*Empathy (QCAE)*	**0.58**	***	**0.20**	***	**0.21**	***	**0.34**	***	**0.21**	***	0.04		**0.34**	***	**0.13**	***
	*Aesthetic reward (AReA)*	**0.39**	***	**0.18**	***	**0.14**	***	**0.35**	***	**0.20**	***	**0.10**	***	**0.18**	***	**0.04**	*
	*Music reward (BMRQ)*	**0.46**	***	**0.16**	***	**0.24**	***	**0.34**	***	**0.13**	***	0.07		**0.33**	***	**0.05**	***
	*Art fluency (AFS)*	**0.37**	***	**0.09**	*	0.06		**0.37**	***	**0.10**	*	**−0.09**	*	**0.10**	***	**0.24**	***
	*Openness to experience*	**0.25**	***	**0.09**	***	**0.08**	***	**0.18**	***	0.03		0.00		**0.27**	***	**0.05**	*
	*Impulsiveness*	**−0.11**	***	0.00		**−0.03**	*	0.05		−0.02		0.03		**−0.11**	***	**0.16**	***
	

* *p* < 0.05

** *p* < 0.01

*** *p* < 0.001.

**Table 4.** Happiness induction results (*N* = 55) from two-tailed paired t-test. Participants rated the “Happy” and “Sad” items we appended to the PANAS mood questionnaire on a 5-point Likert scale from “Not at all” to “Very” happy or sad. Results were re-scaled to have range 10.

Happiness induction results				
*Mood questionnaire item*	*Mean difference*	*t*	*p*	*df*
Happiness	0.58	1.93	0.058	54
Sadness	−0.95	−4.44	< 0.001	54

**Table 5.** Sadness induction results (*N* = 54) from two-tailed paired t-test. Participants rated the “Happy” and “Sad” items we appended to the PANAS mood questionnaire on a 5-point Likert scale from “Not at all” to “Very” happy or sad. Results were re-scaled to have range 10.

Sadness induction results				
*Mood questionnaire item*	*Mean difference*	*t*	*p*	*df*
Happiness	−1.48	−4.66	< 0.001	53
Sadness	1.85	6.21	< 0.001	53

**Table 6.** Neutral mood induction results (*N* = 55) from two-tailed paired t-test. Participants rated the “Happy” and “Sad” items we appended to the PANAS mood questionnaire on a 5-point Likert scale from “Not at all” to “Very” happy or sad. Results were re-scaled to have range 10.

Neutral induction results				
*Mood questionnaire item*	*Mean difference*	*t*	*p*	*df*
Happiness	−0.32	−1.16	0.25	54
Sadness	−0.29	−1.38	0.17	54

## Data Availability

Data is provided within the manuscript file at the following URL: https://osf.io/92vyu/?view_only=33ba830f92954f9db47acc4284fa1f42
